# Hybrid fixation with ESIN for both bone forearm fractures in adults: A case report and literature review

**DOI:** 10.3389/fsurg.2022.949727

**Published:** 2022-07-26

**Authors:** Xiaodong Bai, Zhenyu Liu, Wentao Chen, Baojun Wang

**Affiliations:** Department of Orthopedics, Beijing Friendship Hospital, Capital Medical University, Beijing, China

**Keywords:** forearm fractures, open reduction and internal fixation, elastic-stable intramedullary nailing, case report, hybrid fixation

## Abstract

**Objective:**

Both bone forearm fractures are common in children and rare in adults. The main surgical treatment is open reduction and internal fixation (ORIF) with plate, while the hybrid fixation of elastic-stable intramedullary nailing (ESIN) and the plate has been rarely reported before.

**Case report:**

We report a case of a 29-year-old male patient with both bone forearm fractures. Temporarily closed reduction and plaster external fixation were performed in the emergency room, and the patient was admitted to the orthopedic trauma ward for surgery. The patient underwent open reduction and plate fixation of the ulna and closed reduction and ESIN fixation of the radius. The fractures healing was satisfactory and the internal fixation was removed 12 months postoperatively.

**Conclusion:**

The hybrid fixation of ESIN and plate is an effective option for both bone forearm fractures in adults.

## Introduction

Both bone forearm fractures are technically challenging for orthopedic surgeons (1). The peak incidence rate is mainly for children and adolescents (2, 3), while the incidence rate of adults is low. At present, elastic-stable intramedullary nailing (ESIN) is the main treatment option for both bone forearm fractures in children and adolescents with the advantages of minimal invasion and less damage to fracture blood supply (3). However, ESIN is not stable fixation and patients need to be assisted with plaster external fixation after the operation, resulting in low postoperative comfort and inability to exercise early (4). The main treatment for both bone forearm fractures in adults is open reduction and internal plate fixation (5). There are some problems associated with internal plate fixation, such as soft tissue injury, fracture nonunion, secondary fracture after plate removal and so on. Therefore, we used hybrid fixation in the treatment of both bone forearm fractures in an adult in our hospital. We report a 29-year-old patient who presented with both bone forearm fractures and underwent open reduction and internal plate fixation of the ulna and closed reduction and internal ESIN fixation of the radius.

## Case presentation

**Figure 1:**
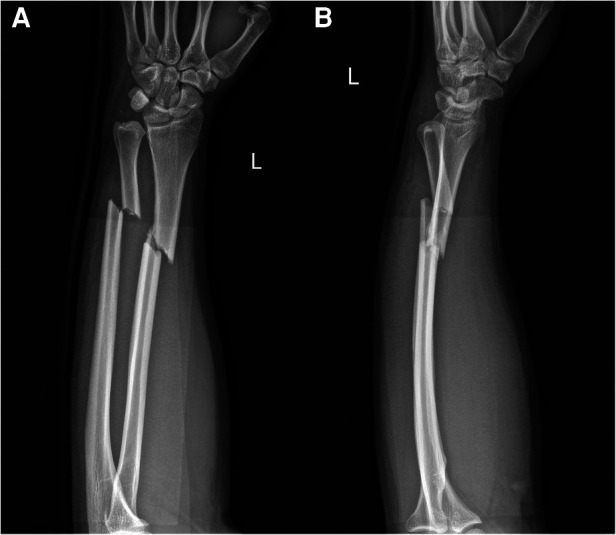
X-ray images (**A,B**) of the left forearm showed both bone forearm fractures with marrow edema.

**Figure 2:**
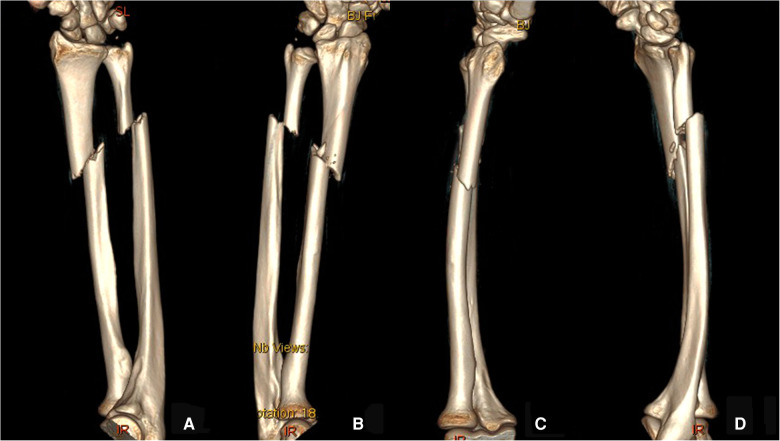
Preoperative three-dimensional reconstructive CT images (**A–D**) of the left forearm.

**Figure 3:**
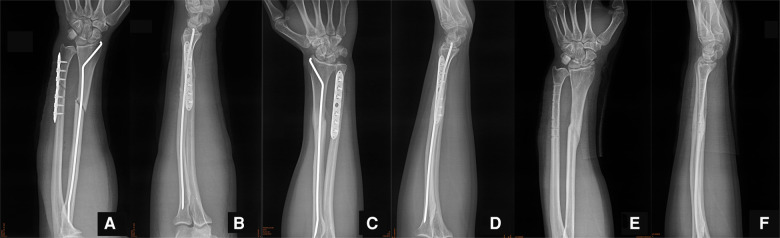
(**A,B**) Postoperative x-ray films of the left forearm. (**C,D**) X-ray films of the patient at 1 month after surgery. (**E,F**) X-ray films of the patient after removal of the internal fixation at 12 months after surgery.

**Figure 4:**
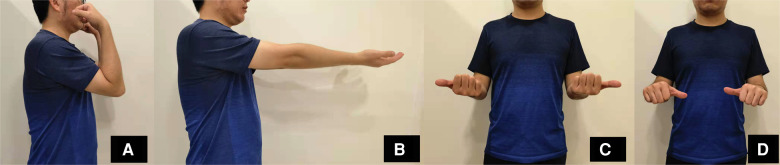
Follow-up after 12 months of surgery of the patient. (**A**) The flexion and (**B**) extension functions of the elbow joint. (**C**) The supination and (**D**) pronation of the forearm.

A 29-year-old male patient fell and suffered trauma to his left forearm while riding a bicycle. He was unable to move his left arm immediately because of extreme pain and was sent to the emergency department. The patient had no family history of osteoporosis or pathological fractures. He had no history of consuming alcohol, smoking, or using drugs. He was 182 cm in height and 95 kg in weight (the body mass index was 28.7 kg/m^2^). Swelling and deformity of the left forearm were found, and the range of motion was restricted. Considering the possibility of both bone forearm fractures, the emergency doctor suggested the patient to take x-ray examinations (Figures 1A,B). X-ray examinations showed both bone forearm fractures on the left forearm. According to AO-OTA classification, the fracture classification of this patient was 2R2A2 and 2U2A2. Indications for surgery were clear and temporary plaster external fixation was performed in emergency. The characteristics of both bone forearm fractures were assessed by three-dimensional reconstructive CT scans (Figures 2A–D). We checked the nerves and artery vessels of the patient at different time points, including when the patient went to the emergency department, after admission, and before surgery, to confirm that the patient had no clear artery injury and clinical manifestations of nerve injury. The patient underwent open reduction and internal plate fixation of ulna and closed reduction and internal ESIN fixation of radius (Figures 3A,B), with no plaster fixation after the operation. The fracture healed 4 months later (Figures 3C,D). The internal fixation was removed 12 months postoperatively (Figures 3E,F). Disability of arm shoulder and hand (DASH) score was assessed at three-time points after surgery. According to the DASH score, 25 points in the 1st month, 13.7 points in the 4th month, and 6.7 points in the 12th-month. During the 12th month follow-up period, the range of supination and pronation was 88° and 85°, which was 92% and 90% of the contralateral rotation, respectively. (Figures 4A–D).

## Discussion

The peak incidence rate of both bone forearm fractures is found in children and adolescents (6). The incidence rate of both bone forearm fractures in adults is relatively low. The low incidence rate of adult fractures leads to few epidemiological studies. The treatment of both bone forearm fractures in children is mainly conservative (7, 8). For cases with obvious fracture displacement, surgical treatment is usually used. Minimally invasive ESIN is the main surgical treatment, while open reduction and internal fixation (ORIF) is rarely used (9). Both bone forearm fractures in adult are usually caused by high energy trauma, and are usually displaced significantly, which often require surgical treatment. At present, the main surgical treatment is open reduction and internal plate fixation, and a few patients were treated with intramedullary nail fixation (10). Hybrid fixation of ESIN and plate has been attempted in the treatment of both bone forearm fractures in children and adolescents, and satisfactory results have been obtained (11, 12). For both bone forearm fractures in adults, hybrid fixation has been rarely reported before. In our case, we tried to apply the hybrid fixation of ESIN and plate to the surgical treatment of both bone forearm fractures in an adult and achieved satisfactory results.

The common mechanism of this fracture pertains to the axial load on the extended hand or the direct impact on the forearm (13). In addition, poor bone quality, caused by osteoporosis, is an important inducement (14). Patients with both bone forearm fractures often show obvious deformities in the appearance of their forearms. Special attention should be paid to whether there is a risk of an open fracture or potential open fracture during physical examination (15). At the same time, we should focus on the potential forearm nerve injury and evaluate the motor and sensory defects of the anterior interosseous nerve, posterior interosseous nerve, and ulnar nerve (16, 17). Meanwhile, an evaluation of blood supply should include the palpation of the radial artery and ulnar artery pulsation and an evaluation of the capillary filling of the fingers, and this should be evaluated several times at different time points. The consequences of the compartment syndrome of the forearm can be disastrous, so we should always be vigilant, even if its incidence is not high (18). In children and adolescents, both bone forearm fractures are often treated non-surgically, which usually helps to achieve good clinical results. However, there are a few successful cases or studies of conservative treatment of both bone forearm fractures in adults (19). The reduction and fixation should be carried out as soon as possible in the emergency department to create conditions for the surgery that follows. Thorough flushing and debridement should also be carried out, and antibiotics should be used to prevent infection in patients with severe open fractures. The wrist joint and elbow joint should be included in plaster fixation to effectively prevent the rotation of the forearm, and the forearm should remain in a neutral or mild supination position, subject to the patient's tolerance levels of pain and comfort levels.

The surgical treatment of both bone forearm fractures in adults includes open reduction and internal plate fixation and intramedullary nail. Open reduction and internal plate fixation are considered to be the gold standard of treatment. For oblique or transverse fractures, the compression plate can achieve the compression at the fracture site and promote primary fracture healing. In long oblique or spiral fractures, the inter block screw compression technology can achieve the satisfactory clinical curative effect through the neutralization plate. Some studies have shown that the combination of radial plate fixation and ulnar intramedullary nail fixation proved to be a better method, which had good rotation stability and restored the radial arch effectively, and achieved satisfactory clinical results (20). Intramedullary nail fixation can shorten operation time and reduce surgical scar (21). However, it should be noted that the intramedullary nails used in abovementioned studies were straight interlocking intramedullary nails, which could not restore the radial arch or the rotation function of the forearm when fixing the radius. The ESIN used in this case was with the prebending design of the radial arch. The radial arch could be effectively restored by three-point contact fixation, so as to recover the rotation stability of the forearm. During the operation, the placement of the radial ESIN could be completed through a surgical incision just about 2 cm in length. The reduction process completed by manual close reduction or percutaneous poking reduction with Kirschner wires was minimally invasive, compared with traditional open reduction. The forearm rotation axis practically intersected the center of the radial head and the base of the ulnar styloid process, at the center of the ulnar head cylinder (22). During forearm rotation, there was a statistically significant difference in ulnar variance between the pronation and neutral views and between the pronation and supination views; nonetheless, rotating the forearm from neutral to supination did not significantly change the measurement of ulnar variance (23). Anterior forearm rotation could be restored only when the axis was effectively stabilized, and ESIN fixation of the radius allowed local micro motion, which could promote callus growth and fracture healing. In addition, this hybrid fixation method could effectively reduce the risk of secondary fracture after internal fixation removal.

## Conclusion

The hybrid fixation method may be an effective option for treating both bone forearm fractures in adults. For the surgical treatment of these fractures, orthopedic surgeons should follow the principle of minimally invasive and individualized treatment to achieve satisfactory clinical results.

## Data Availability

The original contributions presented in the study are included in the article/Supplementary Material, further inquiries can be directed to the corresponding author/s.
